# (Correcting) misdiagnoses of asthma: a cost effectiveness analysis

**DOI:** 10.1186/1471-2466-11-27

**Published:** 2011-05-23

**Authors:** Smita Pakhale, Amanda Sumner, Douglas Coyle, Katherine Vandemheen, Shawn Aaron

**Affiliations:** 1 University of Ottawa, and The Ottawa Hospital Research Institute, 501 Smyth Road, Ottawa, ON K1H 8L6, Canada; 2 Children's Hospital of Eastern Ontario Research Institute, 401 Smyth Road, Ottawa, ON K1H 8L1, Canada; 3 Department of Epidemiology and Community Medicine, University of Ottawa, 451 Smyth Road, Ottawa, ON K1H 8M5 Canada

**Keywords:** Asthma cost, Canadian asthma cost, asthma cost savings, asthma secondary screening, economic analysis, epidemiology of asthma

## Abstract

**Background:**

The prevalence of physician-diagnosed-asthma has risen over the past three decades and misdiagnosis of asthma is potentially common. Objective: to determine whether a secondary-screening-program to establish a correct diagnosis of asthma in those who report a physician diagnosis of asthma is cost effective.

**Method:**

Randomly selected physician-diagnosed-asthmatic subjects from 8 Canadian cities were studied with an extensive diagnostic algorithm to rule-in, or rule-out, a correct diagnosis of asthma. Subjects in whom the diagnosis of asthma was excluded were followed up for 6-months and data on asthma medications and heath care utilization was obtained. Economic analysis was performed to estimate the incremental lifetime costs associated with secondary screening of previously diagnosed asthmatic subjects. Analysis was from the perspective of the Canadian healthcare system and is reported in Canadian dollars.

**Results:**

Of 540 randomly selected patients with physician diagnosed asthma 150 (28%; 95%CI 19-37%) did not have asthma when objectively studied. 71% of these misdiagnosed patients were on some asthma medications. Incorporating the incremental cost of secondary-screening for the diagnosis of asthma, we found that the average cost savings per 100 individuals screened was $35,141 (95%CI $4,588-$69,278).

**Conclusion:**

Cost savings primarily resulted from lifetime costs of medication use averted in those who had been misdiagnosed.

## Background

Over the past 3 decades the prevalence of physician-diagnosed asthma has increased more than 75% in Canada and in the US [1,2]. Studies from Canada suggest that less than 50% of Canadians receive lung function testing before a diagnosis of asthma is assigned by their physician [3,4]. Therefore for most Canadians a diagnosis of asthma is usually made on clinical grounds alone [3,4]. Similarly, underuse of spirometry in the US [5] and Europe [6] in establishing the diagnosis of asthma is well known.

A recent study [7] by our group determined the proportion of obese and normal weight Canadian adults with an incorrect diagnosis of asthma. Subjects were randomly sampled from 8 cities across Canada by random digit dialing. Subjects were included in the study if they were at least 16 years old, if they were obese (BMI ≥ 30) or normal weight (BMI 20-25), and if they identified themselves as having current, physician-diagnosed asthma. Details of the study design, subject selection, and the diagnostic algorithm for establishing the diagnosis of asthma are discussed elsewhere [7]. This study showed that approximately 30% of enrolled subjects with a history of physician-diagnosed asthma did not have asthma after they were objectively assessed with lung function and bronchial challenge testing and after their asthma medications were tapered off. Misdiagnosis rates were similar in both obese and non-obese subjects.

Adult patients who receive a lifetime diagnosis of a chronic disease like asthma experience frequent activity limitation and potential psychological distress [8]. A diagnosis of asthma can mean a personal and economic burden for patients. Recently, a study from the USA showed that the annual adjusted mean incremental total expenditure for asthma in adults was $2077. Prescription medication and physician office visits were the major drivers of this cost, accounting for 38% and 49% of the cost respectively [9]. A detailed analysis of asthma costs in Canada showed that the total cost of asthma in 1990 Canadian dollars was estimated to be $504 to $648 million. The single largest component of direct costs was the cost of drugs ($124 million) [10].

Overdiagnosis of asthma in the general population is an important public health concern. Mislabeling subjects with a chronic illness like asthma could cost health care dollars and also could adversely affect health related quality of life. In addition, a misdiagnosis of asthma may mean that the actual undiagnosed underlying illness remains untreated. Non-treatment or under-treatment of the patient's actual illness could lead to potentially preventable long term complications which might further jeopardize the health status of the patient and further increase costs.

Our study on asthma misdiagnosis included a concurrent prospective economic analysis. Data on resource use were prospectively collected during the study and analysis of these data is presented here. The specific objective of the present study was to determine, using cost-effectiveness analyses, whether a diagnostic strategy to confirm asthma in patients with physician-diagnosed asthma was cost-effective.

## Methods

**Figure 1:**
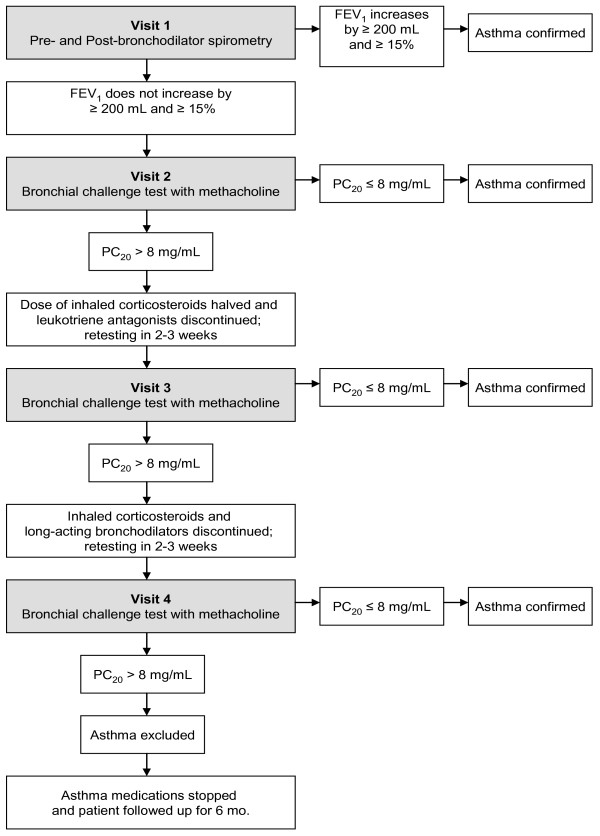
**Serial asthma testing algorithm (Confirmed Asthma = 346, Asthma excluded = 150) (PC**_**20 **_**- the provocation concentration that caused decrease in forced expiratory volume in 1 second (FEV**_**1**_**) of 20%)**.

The present cost-effectiveness analysis is based upon data from the asthma misdiagnosis longitudinal study involving normal weight and obese physician-diagnosed asthmatic subjects [7]. In this study, individuals who reported a physician diagnosis of current asthma were randomly selected from eight metropolitan areas of Canada through random-digit dialing. Subjects with > 10 pack year history of smoking were excluded in order to prevent enrollment of patients with chronic obstructive pulmonary disease (COPD). The individuals then underwent a series of lung function tests and a diagnosis of current asthma was excluded in those who did not have evidence of acute worsening of asthma symptoms, reversible airflow obstruction or bronchial hyperresponsiveness, despite being weaned off asthma medications. Asthma medications were stopped in those in whom a diagnosis of asthma was excluded and their clinical outcomes were assessed over a 6 month period prospectively. Further details of the study design, subject recruitment, and methods are described elsewhere [7]. The asthma diagnostic algorithm used in the study [7] is presented in figure 1. The study was approved by the research ethics boards of the 8 participating study hospitals; economic analysis was part of the ethics application. All patients who participated in the study gave written informed consent.

This analysis focuses on estimating the incremental lifetime costs associated with screening previously diagnosed asthmatic subjects. Analysis was from the perspective of the Canadian healthcare system and subjects were presumed to live a maximum of 50 years from the time of first diagnosis of asthma (although this was varied to 25 years for sensitivity analysis). Costs included the costs of the diagnostic testing algorithm and the lifetime costs of any medication use averted due to misdiagnosis.

### Costs

Healthcare resource use prior to diagnostic screening, during screening and up to six months post screening was collected prospectively for each patient according to the study protocol. The principal resource items of interest were the costs of asthma medications, costs of testing to confirm diagnosis of asthma, costs of emergency room visits, and the cost of a respiratory disease specialist consultation. In a sensitivity analysis, we explored the impact on cost savings if the screening diagnostic algorithm could be managed by a general practitioner rather than by a specialist. The prices of all medications were based on listed prices in the Ontario Drug Benefit Formulary plus the pharmacy markup and dispensing fee^8^. All medications were recorded by drug name, dose, route, frequency and duration, enabling an accurate calculation of costs for each patient. The cost of physician visits and emergency department visits was based on the Ontario Ministry of Health [11,12]. The costs of spirometry testing, bronchial provocative testing and specialist and general practitioner consultation were based on the Ontario Schedule of Benefits 2009 [13]. All costs are presented in 2009 Canadian dollars (tables 1 and 2). The cost of spirometry testing and bronchial provocation testing includes laboratory fee, technician fee, and respiratory specialists' interpretation fee.

**Table 1:** Unit costs (2009 CAN$) for each drug as per the Ontario Drug Benefit Formulary/Comparative Index

Drug Name	Formulation	Cost per puff or pill
**1. Short Acting Beta-agonist**		
**Salbutamol (**Ventolin)	100 mcg MDI, 200 dose pk	$0.08
**2. Long Acting Beta-agonist (LABA)**		
**Salmeterol (**Serevent)	Diskus 50 mch Pd Inh-60 Dose Pk	$1.10
**3. Inhaled Steroids (ICS)**		
**Fluticasone **(Flovent)	250 mcg MDI Inh-120 Dose Pk	$0.78
**4. Cromolyn**		
**Nedocromil sodium **(Tilade)	Puffer, 104 dose Pk	$0.42
**5. Leukotriene Antagonist**		
**Montelukast (**Singulair)	10 mg pill, 30 pills	$3.21
**6. Combination 1 (ICS/LABA)**		
**Budesonide/Formeterol **(Symbicort)	200 mcg/6 mcg Pd Inh-120 Dose Pk	$0.76
**Fluticasone/Salmeterol **(Advair)	Diskus 50/250 mcg Inh-60 Dose Pk	$1.80
**7. Combination 2**		
**Salbutamol/ipratropium bromide(**Combivent)	20 mcg/100 mcg/md Aero 200 Dose Pk	$0.15
**8. Prednisone**	50 mg tab, 30 pills	$0.33
**9. Tiopropium bromide **(Spiriva)	18 mcg Cap, 30 pills	$2.50

**Table 2:** Healthcare and Testing costs for all individuals and asthma medications for individuals for whom the diagnosis of asthma was excluded

Item	Costs per visit/test	Reference
**Physician consultations**		Ontario Schedule of Benefits 2008
Respiratory disease consult	$143.40	
GP Limited consultation	$44.65	
**Emergency Department Assessment**	$187.76	Ontario Ministry of Health: Gaboury 2009
**Spirometry B (Visit 1)**		Ontario Schedule of Benefits 2008
Flow Volume Loop	$28.80	
Repeat after bronchodilator	$8.63	
Total	$37.43	
**Bronchial provocative testing (Visits 2, 3 and 4)**	$80.45	Ontario Schedule of Benefits 2008
**Total Cost of Screening**		
Visit 1 only (11.2%)	$180.83*	
Visits 1 and 2 only (80.2%)	$261.28*	
Visits 1, 2 and 3 only (3.3%)	$341.73*	
All Visits (5.3%)	$422.18*	
Average cost of Screening for all patients	$263.42 (95%CI $259.15 - $267.68)	
Average cost of Screening for Non-Asthmatic patients	$288.10 (95%CI $278.94 - $297.25)	
**Study Medication**	**Number of individuals (%)**	
SABA** as needed only	26 (23.9%)	
SABA daily only	1 (0.9%)	
ICS^# ^as needed only	3 (2.8%)	
ICS daily only	1 (0.9%)	
ICS as needed plus SABA	23 (21.1%)	
ICS daily plus SABA	12 (11.0%)	
COMBO^## ^as needed only	6 (5.5%)	
COMBO as needed plus other medications	13 (11.9%)	
COMBO daily only	2 (1.8%)	
COMBO daily plus other medications	17 (15.6%)	
Leukotriene Antagonist (all combinations)	5 (4.6%)	

* Includes the costs of a respiratory disease specialist consultation

** SABA - Short-acting β-agonist

^# ^ICS - Inhaled corticosteroids

^## ^COMBO - combination therapy with ICS and long-acting β-agonist

### Determination of Incremental Costs of The Diagnostic Screening Algorithm

Based on the results of the diagnostic screening, individuals in the longitudinal study were categorized into three groups: those determined not to be asthmatic, those that had their diagnosis of asthma confirmed, and others (individuals who withdrew before all test were completed and who could not be classified as having asthma or not). For the latter two categories we assumed that diagnostic screening will not affect the future costs associated with asthma. Thus, the incremental costs were solely the costs of the diagnostic screening itself for these two categories.

**Figure 2:**
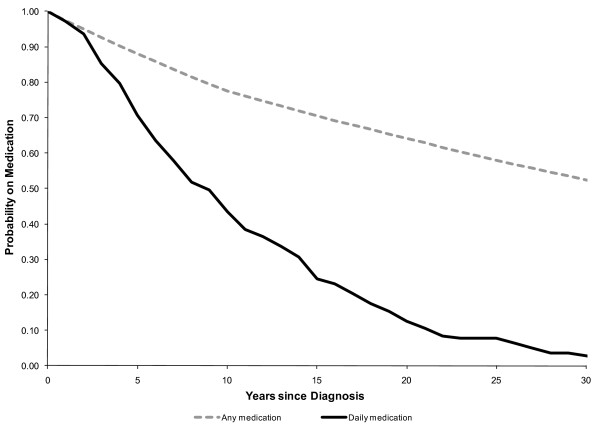
**Years since diagnosis of asthma and probability of being on asthma medication**.

**Figure 3:**
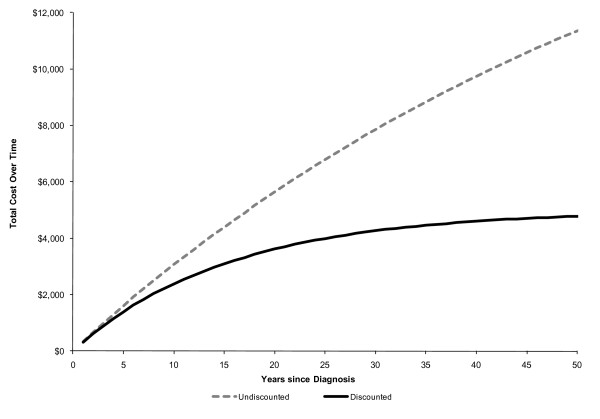
**Discounted lifetime cost of asthma medication by years since diagnosis for the subjects where diagnosis of asthma was ruled out by diagnostic algorithm**.

Those determined not to be asthmatic were followed up prospectively for six months after the conclusion of the diagnostic screening. Resource use that could be associated with asthma care was monitored and was considered an incremental cost associated with the diagnostic screening. For those in whom asthma was ruled out by our diagnostic algorithm, and who were on medication prior to diagnostic screening we estimated the lifetime costs of medication use avoided as a result of the diagnostic screening. This required a four step approach. First, from the longitudinal study we estimated the probability that non asthmatics would be on medication in the year after the diagnosis of asthma using methodology congruent to survival analysis. (figure 2). For the 20 individuals (3.7%) for whom information on the year of diagnosis was missing we used the average years since diagnosis for each group. Secondly, we estimated the cost of medication by year since diagnosis for those in whom asthma was ruled out. This was estimated through linear regression analysis using data from the longitudinal study to adjust for increase in costs of annual medication based on year of diagnosis. Thirdly, we estimated the discounted lifetime cost associated with asthmatic medication for subjects in whom the diagnosis of asthma was ruled out by our diagnostic algorithm (figure 3). The discounted lifetime cost was the product of the probability of being on medication, the cost of medication and the discount factor, for each subsequent year. Finally we allocated the lifetime cost of medication based on the specific year since diagnosis for each subject who had been taking asthma medication, in whom the diagnosis of asthma was eventually ruled out by our testing algorithm.

### Statistical Analysis

Outcome is presented as the incremental costs of screening per 100 subjects screened. Uncertainty around these estimates were obtained through non-parametric bootstrapping whereby numerous estimates of outcomes is obtained by sampling with replacement from the original data set to obtain a new data set of the same size. These bootstrap estimates are assumed to provide empirical distribution of the data which is an adequate representation of the true distribution of the data. Uncertainty is thus represented by the set of estimates obtained from the observed data [14]. A random sample of 100 individuals from the original data from each group was bootstrapped. For each iteration of bootstrapping (n = 5000), we calculated the percentage of individuals who were not asthmatic, percentage of non asthmatic individuals who were on medication and the incremental cost of screening. These outcomes were then averaged over the 5000 bootstrap replicates; means and 95% Credible Intervals (CI) were calculated. Sensitivity analysis was conducted on drugs that were taken as needed (e.g. short-acting bronchodilators); the first approach was to use a low number of inhalations per week (1 to 2 inhalations per week) and the second approach was to use a high number of inhalations per week (4 to 8 inhalations per week). All analyses were conducted using SPSS 16.0.

## Results

### Asthma diagnosis and medications

Five-hundred and forty individuals with physician-diagnosed asthma entered into the study, however 41 patients withdrew prematurely before study completion and three patients were categorized as 'unable to classify' because their baseline FEV_1 _was less than 60% predicted and they were unable to safely undergo a bronchial challenge test. In total 496 individuals completed all of the study assessments and could be conclusively evaluated for a diagnosis of asthma. Of the 496 patients who could be conclusively diagnosed, 346 were confirmed to have asthma and 150 did not have asthma when objectively studied. We were thus able to exclude a diagnosis of asthma in 150 of the 540 subjects who entered into the study (28%; 95% CI: 19-37%). Of these, 109 (73%) were currently taking asthma medications - 37 of these on a daily basis.

The number of years since diagnosis of asthma and probability of being on asthma medication in the cohort of subjects in whom diagnosis of asthma was ruled out is depicted in figure 2. Although the majority of patients began using daily asthma medication at the point of initial diagnosis, less than 50% were on daily medication ten years after their diagnosis date. However, more than half were still on some form of asthma medication on an intermittent basis, even 30 years after diagnosis.

Linear regression analysis was employed to adjust for any increase in the costs of annual medication based on years since diagnosis. We incorporated the increasing annual costs of medication each year (an increase of $1.67 (95% CI: -$6.10 to $9.45) although this increase was small and was not statistically significant. Accumulated costs of asthma medications increased with length of time since diagnosis. Ten years after diagnosis, the discounted accumulated cost of asthma medication was approximately $2000 (Figure 3).

Table 2 shows the cost of screening patients for asthma. The cost of physiological testing at each step of the screening algorithm (figure 1) was $37.43 for the first step and $80.45 for steps 2, 3 and 4. In addition, we assumed each participant would have one respiratory disease specialist consultation (an additional cost of $143.40). Thus, if a patient underwent all the steps in the screening algorithm, the total cost per patient would be $422.18 (Table 2). However, 91.4% of patients required only two or fewer steps of the screening algorithm [15]. Thus, the average cost per patient of the asthma screening algorithm was $263.42 for all patients and $288.10 for those for whom a diagnosis of asthma was excluded. Table 2 also provides the breakdown in asthma medication by individuals for whom a diagnosis of asthma was excluded - this represents the resources that can be saved through exclusion of asthma diagnosis.

### Economic Impact of Screening

On average, 28% (95% CI: 19-37%) of subjects did not have asthma (Table 3). Of these, 71.4% (95% CI: 46.4-100%) were on some asthma medications. Incorporating the incremental cost of screening for the diagnosis of asthma and the forecasted cost savings through identification of patients who were not asthmatics, we found that the average cost savings per 100 individuals screened was $35,141 (95% CI: $4,588 to $69,278) (Table 3).

**Table 3:** Results of non-parametric bootstrapping and sensitivity analysis

Characteristics	Per 100 Patients screened
**% Not Asthmatic**	28
2.5% Credible Interval	19
97.5% Credible Interval	37
**% Not Asthmatic and on Daily Medication**	20
2.5% Credible Interval	13
97.5% Credible Interval	28
**Average Cost Savings**	$35,141
2.5% Credible Interval	$4,588
97.5% Credible Interval	$69,278
*Average cost saving per patient screened is $ 351	
**Sensitivity Analysis**	
**25 year time horizon**	
**Average Cost Savings**	$24,390
2.5% Credible Interval	$1,181
97.5% Credible Interval	$47,600
*Average cost saving per patient screened is $ 243	
**GP consult rather than specialist**	
**Average Cost Savings**	$45,016
2.5% Credible Interval	$14,463
97.5% Credible Interval	$79,153
*Average cost saving per patient screened is $ 450	
**As needed 1 to 2 puffs of short-acting bronchodilator per week**	
**Average Cost Savings**	$29,798
2.5% Credible Interval	-$3,396
97.5% Credible Interval	$102,307
*Average cost saving per patient screened is $ 298	
**As needed 4 to 8 puffs of short-acting bronchodilator per week**	
**Average Cost Savings**	$41,606
2.5% Credible Interval	$474
97.5% Credible Interval	$113,843
*Average cost saving per patient screened is $ 416	

### Sensitivity analysis

Three sensitivity analyses were conducted (Table 3). First, analysis restricted the time horizon to only 25 years. Based on this assumption the average cost savings per 100 individuals screened were reduced: $24,390 (95% CI: $1,181 to $47,600)

Secondly, analysis explored the incremental cost savings if the screening could be undertaken assuming only a GP consultation rather than a specialist consultation. Based on this assumption the average cost savings per 100 individuals screened increased to $45,016 (95% CI: $14,463 to $79,153)

Finally, a sensitivity analysis was conducted on drugs that were taken as needed, for a low number of inhalations per week (1 to 2 puffs of as needed inhaled short-acting bronchodilator medication per week) the average cost savings per 100 individuals screened was $29,798 (95%CI: -$3,396 to $102,307) and for a high number of inhalations per week (4 to 8 puffs per week) the average cost savings per 100 individuals screened was $41,606 (95% CI: -$474 to -$113,843).

## Discussion

This economic analysis of our large multi-center longitudinal study sheds light on the long term economic implications of asthma misdiagnosis. We discounted the impacts of substantial cost savings accumulating over time within the analysis to reflect societal preferences for the timing of events. However, even after discounting we found a significant cost associated with asthma misdiagnosis. Lifetime costs for asthma medications in misdiagnosed patients (who would not be expected to benefit from these medications since they did not have asthma) amounted to over $4000 per patient.

We undertook our study to objectively screen randomly-selected individuals with a previous physician diagnosis of asthma in order to confirm asthma in these individuals. The average cost savings per patient screened was more than $351. In Canada which has a population > 33 million, the prevalence of physician-diagnosed asthma is 8.5%, and more than 2.8 million people have been diagnosed with asthma. If one assumes an asthma misdiagnosis rate of 28-30% then this would imply that over 785,000 people have been mistakenly diagnosed. A secondary screening program to conclusively establish a diagnosis of asthma in those who have been previously diagnosed, such as described in this study, would be expected to remove many mistaken diagnoses and could ultimately generate more than $275 million in cost savings. The saved health care dollars could be redirected to better manage asthma in those who are truly confirmed to have the condition [13]. Though our study is based upon Canadian data, asthma diagnostic practices are quite similar throughout North America [5] or Europe [6]. However the costs of asthma medications in the US are considerably higher than in Canada [16,17].

The major strength of this analysis is that all the drug costs are based on accurate data prospectively collected from subjects rather than estimates. The non-parametric bootstrapping used in this analysis enabled full incorporation of the uncertainty arising from the limited sample size.

Cost analysis is an inexact science; and there are built-in limitations to cost-analysis studies. Measurement errors, uncertainties in the estimates, and recall bias may affect data obtained from such studies - although the direction of such bias is unknown. We have attempted to account for some of these limitations by considering most conservative estimates of costs and by obtaining cost information from multiple sources. In addition, because we did not have data to determine indirect costs; we only analyzed direct healthcare costs in this study.

A systematic analysis recently summarized the economic burden of asthma in developed and some developing countries [18]. Most of these studies are based upon databases [17,19], cross-sectional survey data [10], patient re-call [16,19,20] or estimation of costs [10,17]. All these studies included direct and indirect costs of asthma which included costs of asthma management, asthma medications, asthma related urgent care utilization and absenteeism related cost [21]. None of these studies addressed the issue of asthma misdiagnosis or the costs associated with diagnosing or misdiagnosing asthma.

Establishing a correct diagnosis of asthma even in subjects diagnosed many years ago seems to be a cost effective option. Whether this should be done via a widespread secondary screening program based upon the algorithm utilized in our study or whether it should be done on a case-by-case basis is open to discussion. Organized, secondary screening programs could be a viable option as shown by our study. Our secondary screening program is not onerous for patients; over 91% of patients seen in our study required only a single pre and post bronchodilator spirometry and/or a single bronchial challenge test to confirm asthma [15]. In the long run, such an approach is cost effective considering the direct and indirect costs associated with misdiagnosis. Merely labeling subjects with chronic illness has its toll, as was shown by increased absenteeism in subjects who were made aware of their diagnosis of hypertension even when they were not started on any antihypertensive medication [22].

A potential limitation of our study is that our algorithm does not incorporate costs associated with establishing alternative diagnoses in those patients in whom asthma was ruled out. Our costing algorithm already incorporates a consultation with a respiratory medicine specialist, so the physician cost of making alternative diagnoses is accounted for. However it is possible that once a diagnosis of asthma is excluded, additional testing to confirm alternative diagnoses other than asthma might or might not be required. If this testing occurs, these costs would not be attributable to our algorithm which is designed to rule in or rule out asthma, and not other diseases. Further research is needed to estimate follow-up costs to establish an alternative diagnosis, once the diagnosis of asthma is ruled out.

Our study adopted a 50 year time horizon to measure the cost savings from the reduction in medication. This does not assume that all patients will live a further 50 years - rather it accumulates the cost savings from the point of screening up until death or 50 years whichever comes first. Adopting a lifetime horizon is standard practice in economic evaluations of chronic diseases. However, there is concern that extrapolation for such a long period may be fraught with inaccuracies. Clearly, the shorter the time horizon adopted in our analysis the less the accumulated cost savings. Sensitivity analysis adopting a 25 year time horizon still found significant cost savings providing confidence to our results.

## Conclusion

In summary, we have shown that making a proper diagnosis of asthma is cost effective and cost saving. We performed diagnostic tests on those who had already been diagnosed with asthma in the past. Though we do not know how the diagnosis of asthma was made initially, we have shown that confirming a proper diagnosis of asthma with physiological tests is cost saving since many patients who are found to have been misdiagnosed can be safely discontinued from their asthma medications. In order to avoid the direct medication costs and the indirect cost of carrying a wrong diagnosis of a chronic disease such as asthma, family physicians and specialists need to be encouraged to obtain physiological tests such as spirometry and bronchial challenge tests to correctly diagnose and confirm asthma.

## Competing interests

The authors declare that they have no competing interests.

## Authors' contributions

SP - Design, implementation, interpretation, writing, has had access to and takes responsibility for the integrity of the data and the accuracy of the data analysis; AS - Statistical analysis, interpretation, writing; DC - Statistical analysis, interpretation, writing; KV - Design, implementation, writing; SA - Design, implementation, interpretation, writing. All authors read and approved the final manuscript.

## Pre-publication history

The pre-publication history for this paper can be accessed here:

http://www.biomedcentral.com/1471-2466/11/27/prepub
